# AI-IoT based smart agriculture pivot for plant diseases detection and treatment

**DOI:** 10.1038/s41598-025-98454-6

**Published:** 2025-05-13

**Authors:** Amin S. Ibrahim, Saeed Mohsen, I. M. Selim, Roobaea Alroobaea, Majed Alsafyani, Abdullah M. Baqasah, Mohamed Eassa

**Affiliations:** 1 Electronics and Communication Department, Faculty of Engineering, Ahram Canidian University (ACU), 6 October City, Giza, 12591 Egypt; 2 Department of Electronics and Communications Engineering, Al-Madinah Higher Institute for Engineering and Technology, Giza, 12947 Egypt; 3 Department of Artificial Intelligence Engineering, Faculty of Computer Science and Engineering, King Salman International University (KSIU), El-Tor, 46511 South Sinai Egypt; 4 Faculty of Computer and Artificial Intelligence, Sadat City University, Sadat City, Egypt; 5 Department of Computer Science, College of Computers and Information Technology, Taif University, P.O. Box 11099, 21944 Taif, Saudi Arabia; 6 Department of Information Technology, College of Computers and Information Technology, Taif University, P. O. Box 11099, 21944 Taif, Saudi Arabia; 7 Department of Computer Science, Faculty of Information Systems and Computer Science, October 6 University, Giza, Egypt; 8 Applied Science Research Center, Applied Science Private University, Amman, Jordan

**Keywords:** Artificial intelligence (AI), Internet of Things (IoT), Smart agriculture, Unmanned aerial vehicle (UAV), Pivot, Plant diseases detection, Deep learning (DL), Computational science, Computer science

## Abstract

There are some key problems faced in modern agriculture that IoT-based smart farming. These problems such shortage of water, plant diseases, and pest attacks. Thus, artificial intelligence (AI) technology cooperates with the Internet of Things (IoT) toward developing the agriculture use cases and transforming the agriculture industry into robustness and ecologically conscious. Various IoT smart agriculture techniques are escalated in this field to solve these challenges such as drop irrigation, plant diseases detection, and pest detection. Several agriculture devices were installed to perform these techniques on the agriculture field such as drones and robotics but in expense of their limitations. This paper proposes an AI-IoT smart agriculture pivot as a good candidate for the plant diseases detection and treatment without the limitations of both drones and robotics. Thus, it presents a new IoT system architecture and a hardware pilot based on the existing central pivot to develop deep learning (DL) models for plant diseases detection across multiple crops and controlling their actuators for the plant diseases treatment. For the plant diseases detection, the paper augments a dataset of 25,940 images to classify 11-classes of plant leaves using a pre-trained ResNet50 model, which scores the testing accuracy of 99.8%, compared to other traditional works. Experimentally, the F1-score, Recall, and Precision, for ResNet50 model were 99.91%, 99.92%, and 100%, respectively.

## Introduction

The increasing demand for agriculture goods is continually escalated due to the rapid population growth all over the world^1^. The need for the crop yield production is expected to double its output by 2050. It means that the crop production should be increases by 2.4% each year^2^. However, the crop yield production is lost by about 20-40% every year due to the plant diseases and insect pests. As well as the shortage of water resources required to grow the plant. Plant disease identification has emerged as a critical concern in recent years^3^. Among the variety of fruits and vegetables consumed, tomatoes, potatoes, and bell peppers are particularly significant. Timely identification of diseases affecting the leaves of these crops is critical. Plant leaves serve as the primary means of detecting leaf infections since the majority of disease symptoms manifest visibly on the leaves^4^.

An automated image-based disease detection and classification mechanism is essential for ecosystem health and ensuring food production. Advanced agriculture techniques, such as image processing and DL rather than encountered machine learning (ML) challenges^5^, offer promising approaches for disease identification and management, potentially revolutionizing agricultural practices. They are utilizing advanced technology, analyzing plant images, eliminating reliance on subjective judgments and delivering more reliable results. Timely identification of crop diseases is crucial for taking necessary action, preventing extensive damage in large-cultivated areas.

Adaptive transfer learning technique as a branch of deep learning could solve ML limitations. It can achieve better performance on the target task with less data and computational resources compared to the inefficiencies associated training a model from scratch^6^. This not only reduced the demand for extensive computational resources but also enhanced the model’s ability to generalize well to diverse datasets. That’s why we chose to embrace transfer learning in our study, as it offered significant advantages. In this paper, the transfer learning technique in the DL was used in this paper for the plant diseases detection.

A diverse advanced techniques were released not only to solve the problem of the plant diseases, but extended to preserve water irrigation and mitigate the spread of pests…etc. They should be equipped on agriculture devices as drones, and robotics to particularly handle the farming operations at the agriculture field. Unmanned Aerial Vehicles (UAVs) or drones is becoming a promising development technology toward remote agriculture applications. UAVs are remotely controlled and wirelessly connected using WSN technology to fly over a wide range of field for developing agriculture applications such as plant diseases detection and pest attack control There are more than 250 models over all the world in the agriculture field^7–9^. UAVs may be equipped by RGB camera, or Hyper-spectral camera, or Multi-Spectral camera based on the required agriculture use case to capture aerial images about the cultivated field as an extensive data set to develop the deep learning algorithms for plant diseases prediction, pesticides spraying, and plant growth monitoring^10–12^.

Most of UAV models with its different characteristics and attributes can perform two main agriculture operations: (1) aerial imagery for real time data monitoring of the crop production and the vegetation landscape, (2) spraying liquid “fertilization, chemical pesticides, water” for plant diseases treatment, pest attack control, and irrigation, respectively^13^. However, it is difficult to practically design and implement one UAV model for performing two major operations. The characteristics and attributes “endurance, weight, speed, and cost” should be also considered and evaluated before design UAV model on the basis of the type of the farming operation and their requirements. UAVs have many limitations due to high cost, low battery lifetime, communication distance. Sometimes the flight time is insufficient to develop all farming operations that required from UAV^14^.

On the other side, the human being could be replaced by robotics for a better and smart management of crop yield in the farming operations. Robotics perform a multi-tasking operations to preserve time, human efforts, and cost in the multiple agriculture use cases^15^. They can recognize the suitable time for the water irrigation, the sowing of seeds, weeding, fertilization, spraying pesticides for pest attack, and the plant diseases detection^13,16–18^. However, working robotics in the ground operations causes many limitations such as obstacles, terrain or uneven plains, path planning, speed, and performing multi agriculture tasks with reduced and less hardware equipment^15,19^.

Rather than the works^3–6^, the paper is working to exploit both image processing and deep learning techniques for the robustness plant diseases detection. Both techniques are setup on the proposed hardware pilot for the plant diseases treatment. The hardware pilot is placed on the existing central pivot instead of the use of UAVs and robotics to fill the gap of the limitations of UAVs and agriculture robotics. Imposing both image processing and deep learning techniques with the hardware pilot on a central pivot is called artificial intelligent-internet of things (AI-IoT) smart agriculture pivot.

Rather than UAVs, the proposed AI-IoT smart agriculture pivot can perform both two main agriculture missions that performed by different UAV models and cover different regions of a farm field^13^. The pivot height is near to the crops to easily access the field of tall crops and then spray liquid for plant diseases treatment, pest attack control, and water irrigation. Its pivot has a very long arm that covers a large coverage of cultivated regions to perform most air farming operations of UAVs as aerial imaginary for plant diseases detection without need to be equipped by multi-spectral or hybrid-spectral camera that is expensive and consumes high power. On the other side, a proposed AI-IoT smart agriculture pivot can perform all farming operations at low altitude of about 2 ~ 3 m from the ground to avoid the robotics’ limitations.

In this paper, the proposed AI-IoT smart agriculture pivot performs the plant diseases detection and treatment using both techniques of image processing and DL. Accordingly, the proposed tasks in the plant diereses detection and treatment lead to make a robustness decision by the ResNet50 model, score high accuracy, and minimize the loss of the detection and the treatment of the plant diseases on the pivot itself without the use of the cloud computing or edge computing.

The main contributions of this paper are as follows:

- Proposing an AI-IoT smart system agriculture pivot architecture for plant diseases detection and treatment.
- Design and implementation of a hardware pilot to prove the concept.
- Pre-processing images before training, testing, and validating using a ResNet50 deep learning model.
- Classification the leaf images onto 3-healthy images and 8-unhealthy images using a pre-trained ResNet50.
- Spraying the fertilizations to treat the detected plant diseases.
- Design a mobile application to get the farmers advices and guide lines about the plant diseases detection and treatment methods.

The paper is organized as follows: Section “Related work” tackles the related works. The methodology is presented in Section “Methodology”, including the proposed AI-IoT smart agriculture pivot architecture and a hardware pilot. Experimental setup including data reprocessing, plant diseases detection, plant diseases treatment, and mobile App are described in Section “Experimental setup analysis”. Section “Experimental results” explains the experimental results. Finally, the conclusion and the future works are discussed in Section “Conclusion”.

## Related work

In this section, the previous scientific articles tackled the DL models that widely used for solving the problem of the plant diseases detection. While, other works discussed how to carry the advanced technologies “IoT” and techniques on the traditional central pivot for the plant diseases treatment.

For plant diseases detection, a convolutional neural network model was presented for detecting and identifying plant leaf diseases based on visual data to boost accuracy, generality, and the overall efficiency of training. The experimental results showed that the proposed convolutional neural network-based model outperforms other previous models by a classification accuracy of 99.23%^20^.

Sun et al.,^21^ proposed a convolutional neural network architecture FL-EfficientNet (Focal loss EfficientNet), which was used for multi-category identification of plant disease images. The experiment used the public data set New Plant Diseases Dataset (NPDD) and compared it with three models: ResNet50, DenseNet169, and EfficientNet. The accuracy of FL-EfficientNet in identifying ten diseases of 5 kinds of crops is 99.72%. In^22^, R. Santhana Krishnan and E. Julie proposed an enhanced convolution neural network (CNN) based on a visual geometry group-16 (VGG-16) was used for potato leaf disease classification. The convolution layers of VGG-16 along with the Inception and the SE block were used in this research for classification. This model achieved the highest classification accuracy of 99.3%.

- Jerome et al.,^23^, discussed a DL based assessment-based convolutional neural network (A-CNN) method to detect healthy and unhealthy plant leaves. This method was implemented to detect widespread plant diseases and achieve a high accuracy of 92% with the proposed classification. In^24^, T.-Y. Lee, et al., presented a high-efficiency CNN architecture tailored for potato disease detection. The authors developed a training set using image processing techniques. They utilized the Adam optimizer and cross-entropy for model analysis, with a Softmax serving as the final judgment function. This CNN achieved an impressive accuracy of 99.53%.
- Agarwal, et al.^25^ developed a CNN model for potato crop disease classification. The architecture comprised of four convolution layers with 32, 16, and 8 filters in each respective layer, and achieved an impressive accuracy of 99.8%. In^26^, M. Umer, et al. tackled a five-layered CNN model for automatic detection of plant disease utilizing leaf images. For the better training on a CNN model, 20,000 augmented images are generated to perform the accuracy of 99.99%. M. S. Hossain, et al.^27^ proposed the CNN model to obtain an accuracy of 99.44% for training, 97.34% for validation, and 96.88% for testing. For the plant diseases treatment, it is required to carry DL models that aforementioned above or other DL techniques over a hardware pilot, located on a traditional central pivot.

Recently, a central pivot was invented as a mechanical and electrical hardware pilot for water irrigation in an efficient manner. Its technique relied on irrigating water quite uniformly. A pivot sprayed the equal quantity of water over the farm land. Accordingly, Pivot was more suited for irrigating a circular farm land. It had a control unit (CU) to irrigate the field using sprinklers by the same water quantity and the same flow rate. In^28^, A. Shilpa et al., the IoT technology was utilized to develop a traditional pivot version, called center pivot irrigation (CPI), a class of self propelled irrigation system to detect the percentage of water irrigation for each segmented region inside a circular farm land according to the variety of topography and types of soil. The CPI was used for all uneven topographic fields and of course there was no crop limitation by following this system for irrigation. The convenient circular irrigation can cover about 76% of the field, while placing the end gun in the system can increase more additional irrigated area in the field and reduce the unirrigated area. IoT with the Self-Propelled CPI System technology was proposed to monitor the values of temperature and soil moisture in the farm and uses the internet gateway to send the data onto the database for storage, analyzing, and decision making to actuate an appropriate choice whether, irrigation the water or not.

For both water irrigation and fertilization without plant diseases detection, Badreldeen et al.^29^ updated the central pivot system (CPS) using an IoT technology by adding the rain sensor and humidity sensor to the system to improve water irrigation management and save water resources.

For crop irrigation monitoring, Matilla et al.,^30^ presented center pivot irrigation monitoring systems based on the IoT technology and two low-cost communication technologies LoRaWAN and GPS to report on demand the pivot states or change in its operation such as an unexpected stop and monitor the pivot sensors onto the farmer. Both wireless technologies solved the long communication problem that expected in the wide space of field and the far fields for successful irrigation monitoring system. The proposed system electrically explained the interfacing between LoRaWAN and GPS and the CU of the pivot and given a case study to prove the concept. The packet loss, received packets was evaluated to study and analyze an effect of these parameters on the operation of the system.

O. Debauche et al.,^31^ proposed a multi agent system (MAS) based on Kubernetes and Docker architecture^32^ to adapt AI algorithms on heterogeneous clusters at the edge level for computing water use and videos of plant diseases near the pivot and minimize the processing time required to make the decision based rule-conditions.

Several AI algorithms were tested on water requirements and plant village database to validate the operation of the proposed architecture. However, the pest detection wasn’t considered in these works. Table 1 shows a state-of-the-art literature review for previous studies.

**Table 1:** Summary of state-of-the-art literature review.

Reference	Methodology	Dataset	Strengths	Limitations
^21^	Proposed FL-EfficientNet CNN utilizing Neural Architecture Search	10 diseases across 5 crop types	Fast convergence (4.7 h for 15 epochs), effective for real-time applications	There is no mobile deployment
^22^	Enhanced CNN based on VGG16, Inception blocks	Datasets from Kaggle (DataSet 38, Plant Village)	Superior adaptability, automated disease recognition	There are no applications to explore outdoor conditions
^23^	Developed A-CNN with OGWF for preprocessing and feature extraction	Kaggle repository dataset	High sensitivity and specificity, reduced computational demands	It needs evaluation of robustness in diverse conditions
^24^	CNN architecture with Adam optimizer	Database of over 2,000 images	Practical approach for disease recognition	Limited dataset diversity
^25^	CNN with four convolutional layers	3000 training images, 500 test images	Robust model enhancing crop management	Need for standard diagnostic tools in underdeveloped regions
^26^	Optimized five-layered CNN with augmented image dataset	Generated dataset	Outperformed existing methods, practical for real environments	It requires testing in diverse agricultural settings
^27^	CNN developed from Plant Village dataset	2080 bacterial-infected, 1,881 healthy leaves	Enhanced detection efficiency	It has overfitting with smaller datasets
^28^	Focused on Self-Propelled Pivot Irrigation System	–	Advocated for modern irrigation techniques in India	Need for cost-effective solutions for farmers
^29^	IoT-based smart irrigation system with ESP32	–	Reduced water waste, optimized fertilizer application	Requires further scalability studies
^30^	Proposed monitoring systems using GPS and LoRaWAN	Tested in maize crops	Effective data management leveraging cloud services	Challenges with variable packet loss and GPS errors
^31^	Multi-Agent System (MAS) integrated into Edge AI-IoT architecture	–	Improved water management, enhanced pest control	Scalability and image preprocessing enhancements needed

## Methodology

**Fig. 1:**
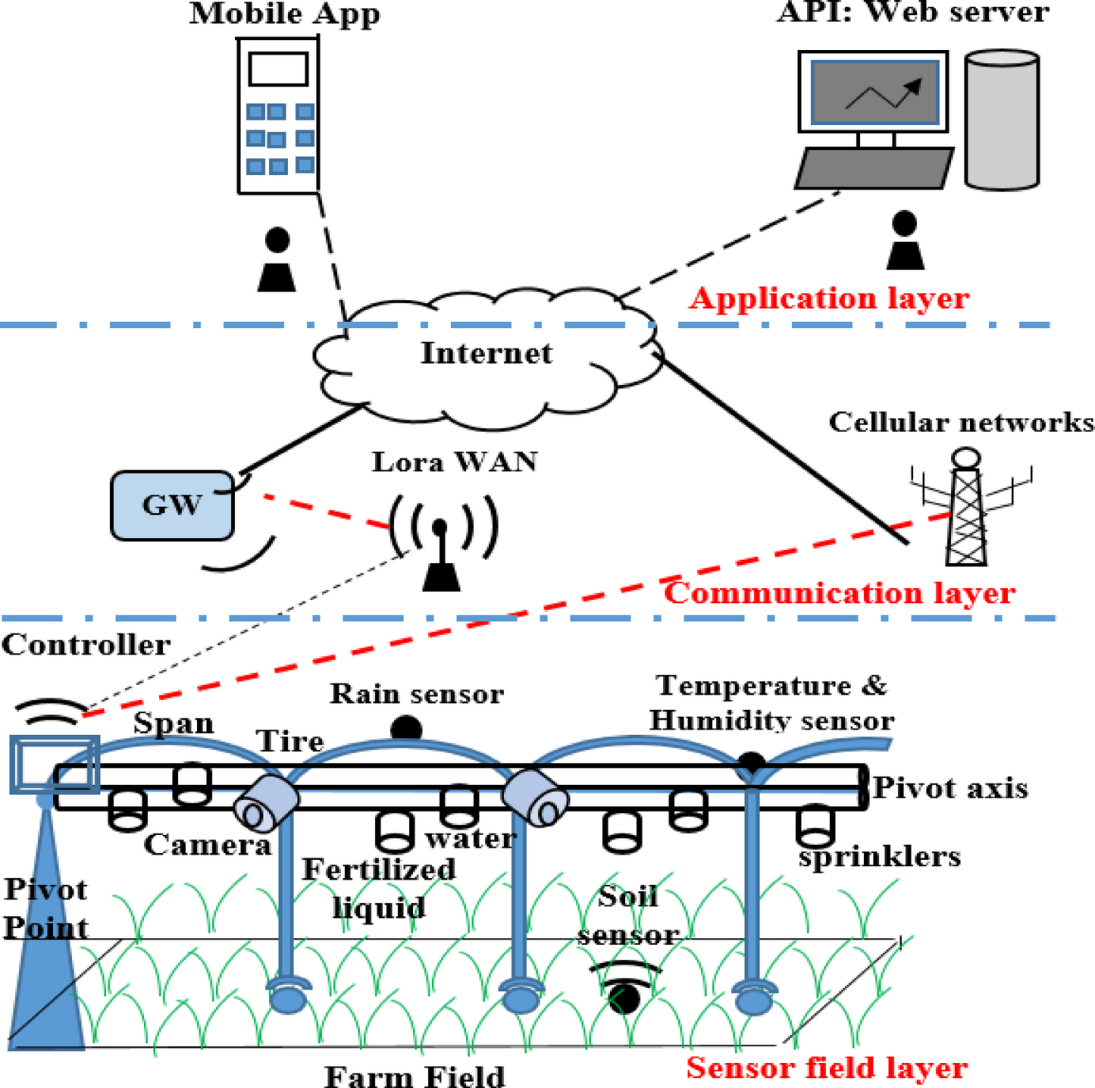
The proposed AI-IoT system architecture.

The proposed work includes: the AI-IoT system architecture and the hardware pilot. The proposed AI-IoT smart system agriculture pivot for plant diseases detection and treatment is shown in Fig. 1.

### The proposed system architecture

The proposed system architecture consists of three major network layers: sensor field layer, communication layer, and application layer. The sensor field layer includes all hardware components associated with the traditional central pivot in the farm field such as controller, sensors, actuators, cameras…etc. The sensor field layer is the physical layer that performs both monitoring and actuating processes in the farm field. The monitoring process is to monitor the real time data about the farm field from the sensors and cameras such as temperature, humidity, rain, soil moisture, and images of a leaf plant. While, the actuating process is to control and manage the overall hardware components by the actuators “sprinklers” for decision making. Such as water irrigation, pest attack control, and plant diseases detection. The actuation process may be operated in the application layer.

The communication layer represents the connectivity between the sensors and cameras associated with the central pivot in the sensor field layer and the user interface in the application layer using the internet cloud. The communication is happened by two ways. The central pivot could be connected directly onto the cloud using the convenient cellular communication networks in one way. It can use an indirect wireless connection using the Lora WAN/Wi-Fi technologies with the Gateway in the other way. The application layer includes the web browser and the mobile App. The web browser can store and log all data sent from the sensor field layer based on its database “MYSQL”. It can also retrieve the log data from the database onto the user interface “API: web browser” to graphically display these data with time and use the statistical analysis to discover a new and useful information about the farm field status. While, the mobile App can periodically receive a real time data monitoring from the sensors and cameras in the sensor field layer. The mobile App assists the farmer for monitoring and controlling the farm field. The mobile App helps the farmer to remotely monitor the status report information about the plant diseases in the farm field and treatment methods such as regions of plant diseases and healthy plant in the farm, the dates of irrigation, dates of spraying liquid of fertilizations, dates of turning ON and OFF the sprinklers…etc. In addition, it enables the farmer to manually control the proposed pivot that located in the sensor field layer by actuators.

### The proposed hardware pilot

Rather than previous works^28–32^, the proposed hardware pilot is designed and implemented on the central pivot itself as part of the sensor field layer of Fig. 1 for the plant diseases detection & treatment. According to the proposed system architecture, the hardware pilot consists of three main dependent levels: physical level, communication level, and network level.

#### Physical level

As shown in Table 2, the proposed pivot includes controller, actuators “two pipelines, sprinklers, water pump”, camera, and communication technology “SIM800L GSM/GPRS Module”. The central pivot is equipped by two pipelines; one pipeline for the use in the fresh water irrigation and the other pipeline for spraying fertilizations on regions, where suffer from plant diseases. Two peer pipelines along the pivot axis are ended by the doubled sprinklers to obtain both water irrigation and spraying fertilizations.

In this proposal, cameras should be deployed at different places of the pivot axis " pivot point, pivot tires, and the pivot end” to cover all regions of the field farm. Cameras are placed on the low altitude of the central pivot to avoid the limitations of the robotics aforementioned above^15,19^. RBG camera " raspberry pi camera” is sufficient to preciously capture images about the plant leafs without the use of a very expensive spectral or hybrid-spectral cameras that equipped on multiple drones^10–12^. The cameras are connected with the Raspberry pi controller, located at the tower of the pivot point.

**Table 2:** The proposed hardware pilot equipment^33,34^.

Hardware pilot equipment	Commercial name	Specifications
Controller	Raspberry pi module	Raspberry Pi 4 Model B 4GB RAM, Cortex-A72 64 Bit Wi-Fi Bluetooth (4GB RAM)
Actuators	Water pump	Water Pump12Vdc Ultra-Quiet Brushless 240 L/H
	Sprinklers	N/A
	Pipelines	N/A
Camera	Raspberry pi camera	Raspberry Pi 4 Camera Module 5MP 1080p OV5647 Sensor Video Webcam for Raspberry Pi Model A/B/B+,Pi 2 Raspberry Pi 2.3,3B+
Communication technology	SIM800L GSM/GPRS module	FM radio broadcast
		Baud rate:1200bps-115200bps
		Call bands: GSM850/EGSM900/DCS1800/PCS1900
		Data: GPRS messages

The CU of the convenient central pivot is replaced by the Raspberry pi controller to perform all irrigation operations followed in the traditional pivot^30^, inspect the plant crop for plant diseases detection, and spray the required fertilization liquid for plant diseases treatment.

Raspberry pi controller can support most of Machine Learning (ML) and Deep Learning (DL) models in the AI technology. Raspberry pi module is a good developer for multiple open source python libraries “Scikit-learn, Py-Torch, Keras, and Pandas” to create the ResNet50 model as one of residual DL models, installed in the Raspberry pi module to train, test, and validate the huge amount of leaf plant images form the NPDD source and obtain the plant diseases detection.

#### Communication level

The role of the Raspberry pi controller is extended to the communication layer in the proposed architecture by the act of its communication unit, called communication level. In this level, the raspberry pi is equipped by different communication technologies “Ethernet, Bluetooth, GSM/GPRS module, and Wi-Fi”, which enable it to remotely communicate with the application layer through the internet. Each communication technology was selected based on its communication range. The proposed hardware pilot is developed by the SIM800L GSM/GPRS module as one of several wireless technologies, widely used for a long distance. It uses SIM800L GSM/GPRS module to send the classified images onto the mobile App at the application layer based on the existing cellular network towers^33^.

#### Application level

The mobile App was setup in this work using flutter framework and python code to diagnose some types of healthy or diseased leaf plant of pepper, potatoes, and tomatoes. As well as, the mobile App can classify among different leaf plant diseases based on the ResNet50 DL model that built in the Raspberry pi controller.

Raspberry pi can interface with camera to perceive leaf plant images in the physical level. The collaborated images were augmented using to extract their important features before the classification process. The augmented images were trained, validated, and tested using ResNet50 model to identify the leaf plant, whether, healthy and diseased for the plant diseases detection. In the communication level, Raspberry pi controller exploits its SIM800L GSM/GPRS module to transfer the classification type “healthy/diseased” onto the mobile App in the application layer.

## Experimental setup analysis

The hardware pilot of the proposed AI-IoT smart agriculture pivot is experimentally setup and operated in this section for the plant diseases detection and treatment, including the following processes: Data augmentation, plant disease detection, plant diseases treatment, and design mobile application.

### Data augmentation

The captured images need to be reprocessed for obtaining a very precise and clear image features extraction before going to the plant diseases classification. The augmentation process grants the ResNet50 classifier model an ability to learn the incoming image features with a very high accuracy and a lowest loss of the plant detection. Images are enhanced using the following image processing techniques: rescaling, shear range, zoom range, horizontal flip, and resizing. Rescaling process is to normalize the image pixel values from the range of 0 to 255 onto the range 0 to 1 by dividing each pixel value by 255.5 in order to make the training of the classifier model is more fast convergence. For example, if the original pixel values of a captured image are: [120, 105]. Thus, the rescaling pixel values become [0.471, 0.412].

**Fig. 2:**
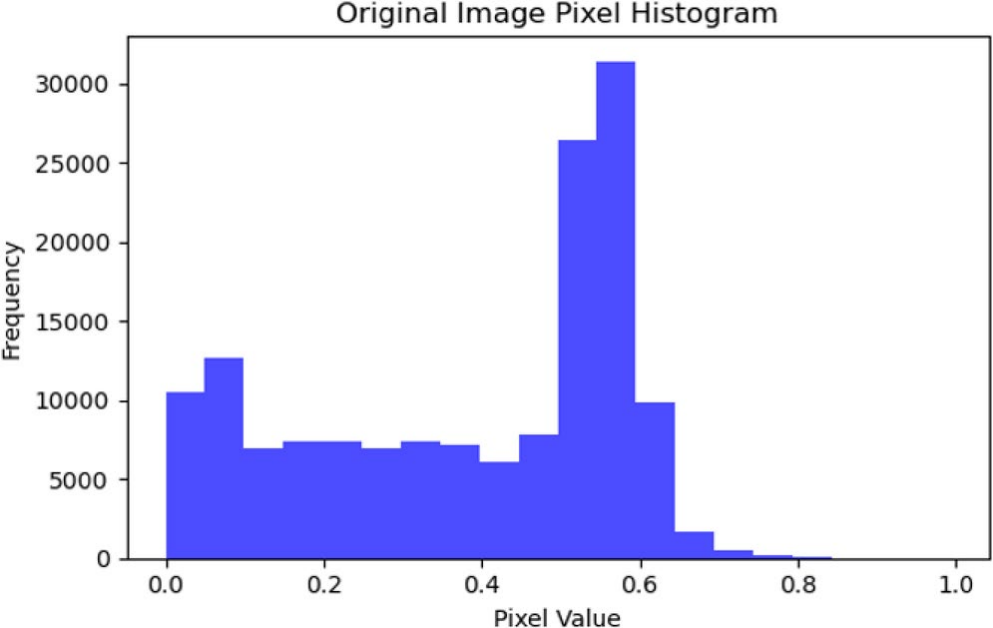
Original image pixel histogram.

**Fig. 3:**
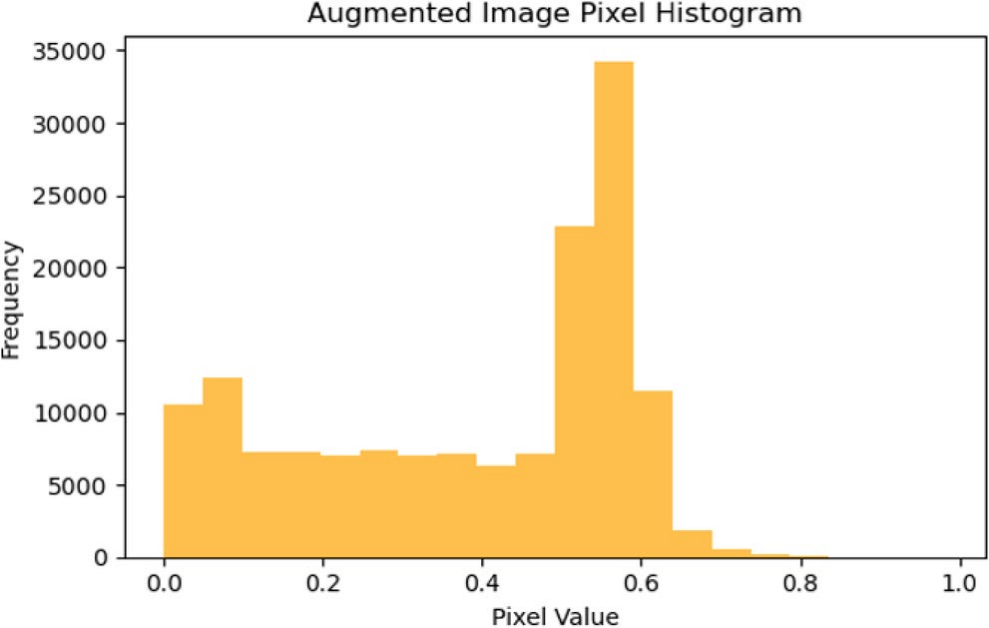
Data augmentation using shearing by range of 0.2.

Shearing process is a transformation that displaces each point in a fixed direction, effectively skewing the image. A shear range of 0.2 means the image will be randomly sheared by an angle up to 0.2 radians (approximately 11 degrees), introducing variability that can make the model robust to such transformations in real-world scenarios. This process represents the random zoom of about ± 20% from the image size. It enables the learning model to recognize objects in the images regardless of variations in their size, simulating a closer or further away camera effect. Figure 2 illustrates the original image pixels’ histogram, while Fig. 3 shows the histogram of the shearing of the image pixels by 0.2 range, showing its impact on the training dataset, compared to the original image pixels.

Horizontal flipping makes the random horizontal flipping of images. It is a common augmentation for scenarios where the orientation of the objects in the images (left to right or right to left) does not imply different categories. It effectively doubles the variety of horizontal orientations the model will encounter, enhancing generalization. A process of resizing all images from a resolution of 256 × 256 pixels to a uniform resolution of 224 × 224 pixels as specified by the target_size parameter for a consistent shape for the image pixels, which is required by the model for classification process. The used dataset is available on the Kaggle repository: https://www.kaggle.com/datasets/vipoooool/new-plant-diseases-dataset.

### Plant diseases detection

A robust deep learning model for plant disease classification is developed by Raspberry pi controller upon data features that are extracted from data augmentation of the images. We employ ResNet50 deep learning model on Raspberry pi controller to analyze plant leaves and diagnose the disease type on the leaf, offering valuable tools for the plant growth in agricultural field. ResNet50 model has emerged as a powerful deep learning model due to the following aspects: (1) its ability to learn complex patterns and images from data, (2) its deep residual learning framework, which facilitates the training of deeper networks without the degradation problem, (3) utilizing ResNet50 pre-trained on the ImageNet dataset exploits its rich feature extractions derived from a vast and diverse set of natural images, making it an ideal starting point for the plant diseases detection, (4) its transfer learning attribute allows ResNet50 model during pre-trained on large-scale datasets to make the fine-tuning for specific tasks, and adaptable to diverse image classification problems.

#### The proposed ResNet50 model structure

**Fig. 4:**
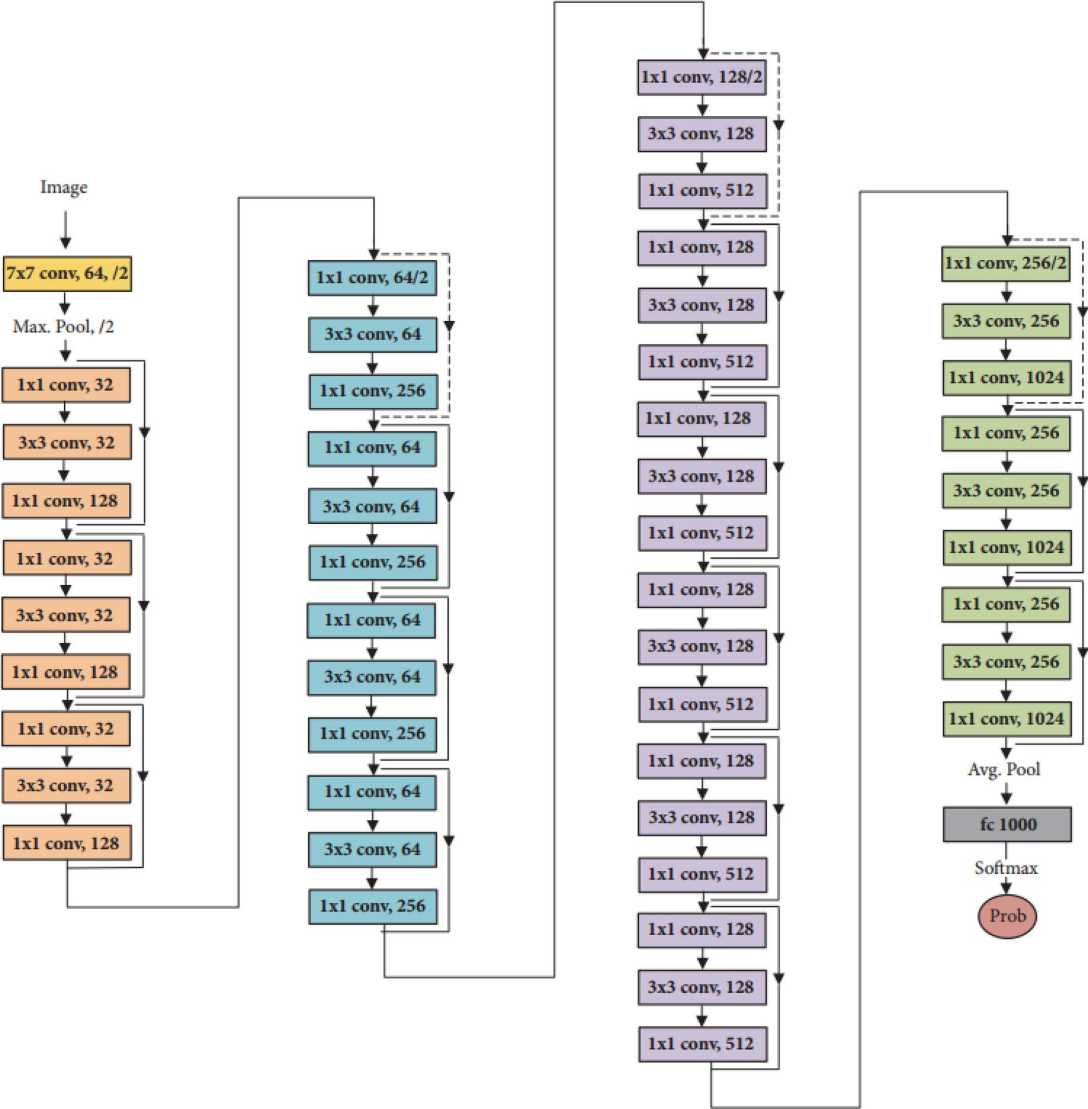
The proposed ResNet50 deep learning model structure^35^.

As shown in Fig. 4, ResNet50 model is structured form 50 layers, including a series of convolutional, pooling, and fully connected layers^35^. A series convolutional layer is attributed as the residual blocks that have shortcut connections to allow the training in the deep neural network to learn residual functions by addressing the vanishing gradient problem. The output of a residual block can be mathematically described in Eq. (1)^36^.

where *x* is the input to the residual block, F(*x*,*W*) represents the operations performed within the block, typically consisting of convolutional layers, batch normalization, and activation functions, W denotes the weights associated with the convolutional layers within the block. The input x is added to the output of the function F(*x*,*W*) to characterize the “residual” nature of the block, enabling the network to learn how to identity functions effectively and thus deeper feature representations without degradation.

1 $$\:\varvec{y}=\varvec{F}\left(\varvec{x},\varvec{W}\right)+\:\varvec{x}$$

After the convolutional layers, ResNet-50 typically uses global average pooling (GAP) layer to reduce each feature map to a single number by calculating the average of the elements. It can minimize the spatial dimensions of the input feature maps, thus summarizing the essential statistical features in each channel. Equation (2) represents the mathematical formula of the GAP layer as follows^37^:

2 $$\:GAP\left(x\right)\:=\:\:\frac{1}{H\times\:W}\sum\:_{i=1}^{H}\sum\:_{j=1}^{W}{x}_{ij}$$

Where *H* and *W* are the height and width of the feature map, respectively, and $\:{\text{x}}_{ij}$ represents the value at the (*i*, *j*) position in the feature map. After the GAP layer, a fully connected layer effectively uses the summarized or reduced features from the GAP to produce the final detection, followed by a softmax activation function for selection and classification among multiple output classes. This layer acts as a classifier or a detector that maps between one of the different reduced features of the plant image in one side and one of multiple output classes in the other side. The output of the fully connected layer was denoted in Eq. (3)^38^.

3 $$\:z\:={\text{W}}_{fc}\:y\:{b}_{fc}$$

Where represents the output of the GAP layer, serving as the input to the fully connected layer, *W*_*fc*_ denotes the weight matrix of the fully connected layer, and *b*_*fc*_ is the biases associated with the fully connected layer.

#### ResNet50 model programming

ResNet50 model as a classifier could be adapted, trained, validated, and tested using python code at the Raspberry pi controller for plant diseases detection and classification. Adaptive ResNet50 model requires the initialization, and adding extra layers instead of the fully connected layer in the top of the ResNet50 structure. Table 3 lists the initialization parameters of the ResNet50 model, and the extra layers added to the original ResNet50 model structure, respectively. About 25,940 images from New Plant Diseases Dataset are trained, validated, and tested for the plant diseases detection.

**Table 3:** Adaptation of ResNet50 model classifier.

Adaptation processes	Parameters	Settings
Initialization	Include_top	False
	Input_shape	(244, 244, 24)
	Weight	ImageNet
	Global	2D Layer
	Average Pooling	Pool-size = 2 × 2
Adding extra layers	A Dropout Layer	Rate: 0.2
	Two Dense Layers	Activation: ReLU
	Final Dense Layer	Activation: Softmax

The dataset comprises to 11-classes; 8-diseased plants and 3-healthy plants as depicted in Table 4. The dataset is split into: 70%, 20%, and 10% for training, validation, and testing, respectively. 18,158-images, 5,188-images, and 2,594-images are specified for training, validation, and testing, respectively. Each group of images involves 11-classes of plants. Table 5 lists the settings of the training parameters.

**Table 4:** Types of plant classes in ResNet50 classifier.

Plant classes	Names
Healthy plant	Pepper, bell__healthy Potato__healthy
	Tomato__healthy
Diseased plant	Pepper, bell__Bacterial_spot
	Potato__Early_blight
	Potato__Late_blight
	Tomato__Bacterial_spot
	Tomato__Early_blight
	Tomato__Late_blight
	Tomato__Tomato_Yellow_Leaf_Curl_Virus
	Tomato__Tomato_mosaic_virus

**Table 5:** Configuration of the training parameters using the Python code.

Training parameters	Value
Epochs	30
Batch size	128
Steps for epoch	142
Optimizer	Adam
Early stopping	val_accuracy, patience = 3

### Plant diseases treatment

After the plant diseases detection and classification process happened, ResNet50 model is able to discover the status “healthy/diseased” of any new captured images by a camera. In case of a new captured image is classified onto unhealthy image, Raspberry pi exploits the actuators “water pump, sprinklers, two pipelines, water tank, and fertilization liquid tank, and switches” for the plant diseases treatment. As shown in Fig. 3, Raspberry pi controller is connected to a water pump motor and the electronic switches to control the process of the plant diseases treatment. It operates the water pump to pull down the liquid from the fertilization liquid tank to its specified pipeline and opens the sprinklers by electronic switches to spray this liquid across the farm regions that are detected as a diseased plant. Otherwise, if the new captured image is detected as a healthy image, the raspberry pi turns off the water pump and close the sprinklers by the electronic switches “solenoid valve”. Table 6 summarizes the work flow of the plant diseases treatment process using Raspberry pi controller.

**Table 6:** Plant diseases treatment cases by raspberry PI.

Plant diseases treatment	Captured images	Water pump	Sprinklers
Case 1	Unhealthy	ON	ON
Case 2	Healthy	OFF	OFF

**Fig. 5:**
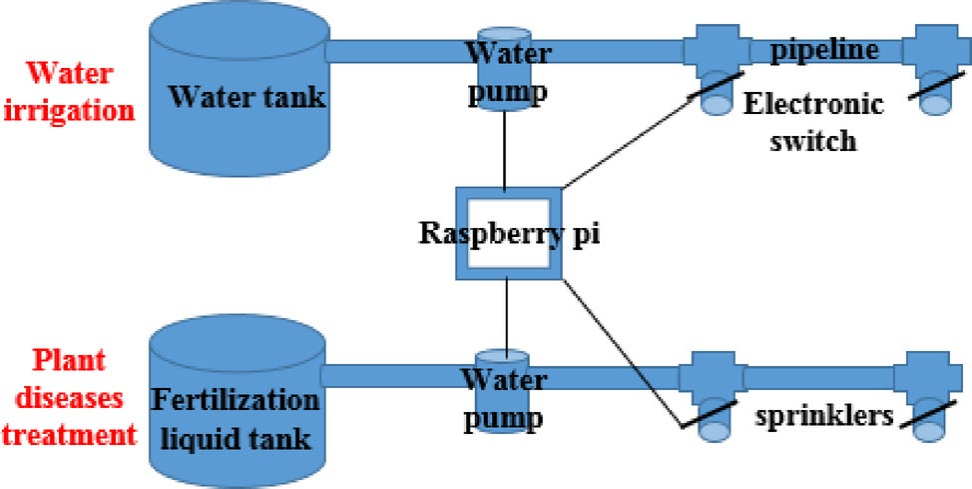
Plant diseases treatment using Raspberry pi controller.

On the other hand, Raspberry pi controller acts as the traditional CU of the central pivot for the water irrigation process. Figure 5 shows the interfacing among the Raspberry pi, sprinklers, and the water pump to water the plant based on the scheduled and frequent irrigation periods, and the type of crop. As shown in Fig. 5, Raspberry pi controller operates the water pump to pull down a fresh water from the water tank onto its specified pipeline and opens their sprinklers by the electronic switches to water the farm land according to the water quantity and irrigation periods, which are required to grow the plant. Otherwise, it turns off the water pump and the sprinklers.

### Mobile application

**Fig. 6:**
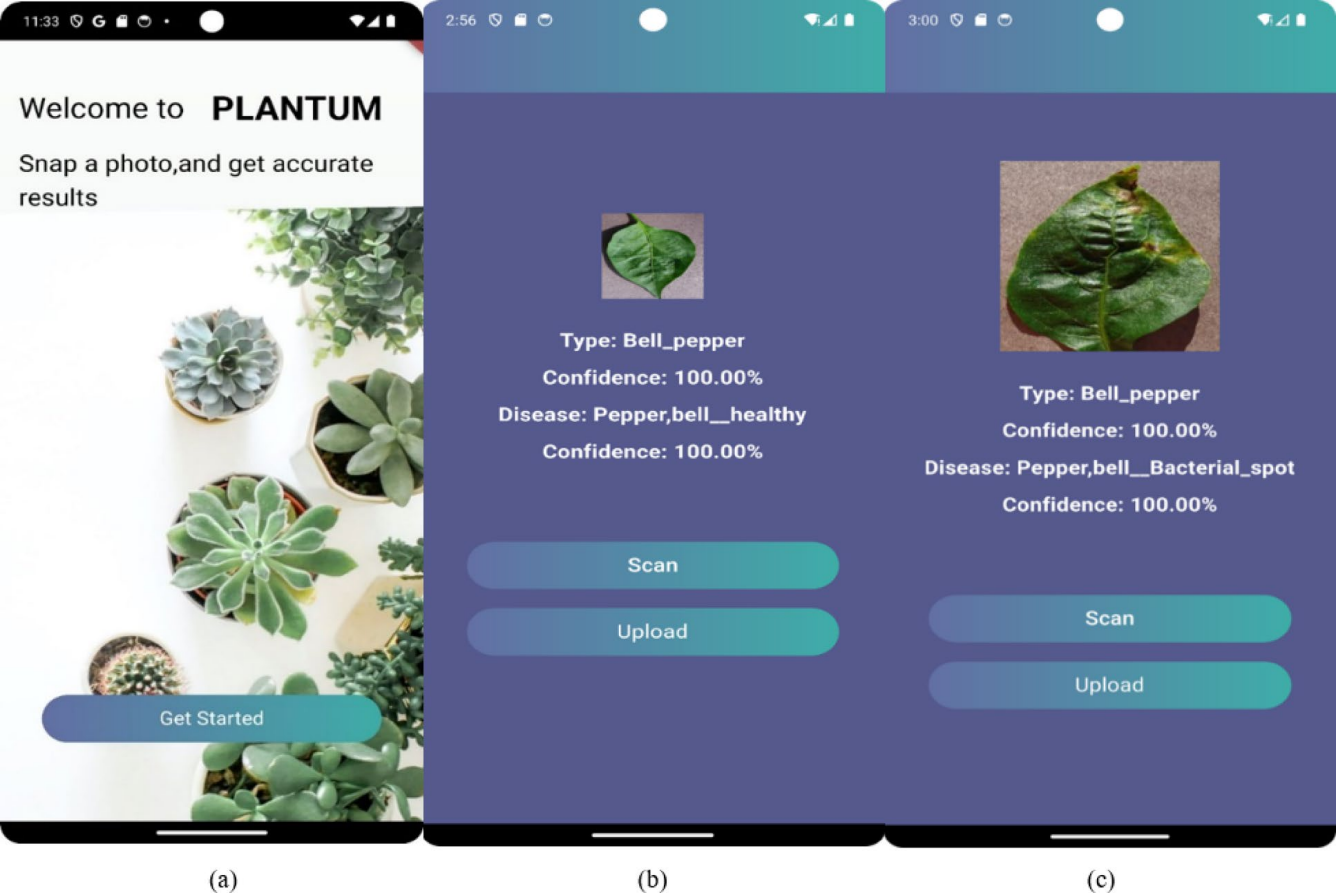
The PLANTUM App for plant diseases detection: (**a**) Main page, (**b**) scanning healthy image, (**c**) Scanning diseases image.

Figure 6a shows the front end page of the main PLANTUM mobile App that was implemented in the application layer of the proposed AI-IoT smart agriculture pivot. The PLANTUM mobile App enables the farmer to test the plant’s leaf whether healthy or diseased, specifically pepper, potatoes, tomatoes. It can also identify the type of plant diseases using the scan operation or the upload operation. In the scan operation, the App can scan any real images of the leaf and classify them onto one of the 11-classes “3-healthy or 8-diseased” as shown in Fig. 6b,c, respectively. Figure 6b shows the healthy bell pepper image after that scanned by PLANTUM App. Firstly, the image is captured by App then retrieved to the Raspberry pi controller in the sensor field layer for testing the plant diseases classification by using ResNet50 model.

The ResNet50 model classifies the captured image onto a healthy bell pepper. This classification is transferred onto the PLANTUM App to carry out this type of classification onto the farmer. Figure 6c describes the diseased bell pepper “bacterial spot” image that scanned by PLANTUM App. The scanned image begins to be sent onto the Raspberry pi controller for classification. After that, the ResNet50 model detects the bacterial spot on the leaf as one of 8-diseased classes. This result is sent onto the PLANTUM App. The upload operation is similar to the scan process. In the upload operation, PLANTUM App can upload any plant images form agriculture websites for plant diseases classification.

After building the proposed hardware pilot in our experimental lab model, the processes of data augmentation, plant diseases detection, plant diseases treatment and mobile App were cooperated together to develop the operation of the proposed hardware pilot as follows:

- Monitoring the images of the leaf plant in the farm field that captured from a camera on the raspberry pi.
- Augmentation images “rescaling, shearing, zooming, flipping, and resizing” to extract their precise features using python code on the Raspberry pi controller.
- Image classification “plant diseases detection process” as one of 11-classes of the leaf plant using ResNet50 model that adapted, configured, and programmed through python code on raspberry pi controller.
- Controlling the central pivot “plant diseases treatment process” by an act of Raspberry pi controller and actuators “sprinklers, pipelines, water tank, electronic switches”.
- Figure out the type of leaf plant classification from Raspberry pi controller onto the PLANTUM Mobile App using SIM800L GSM/GPRS module.

## Experimental results

Both data augmentation and the plant diseases detection processes were analyzed and evaluated in the experimental setup section to obtain an optimum plant diseases detection.

### Data augmentation results

**Fig. 7:**
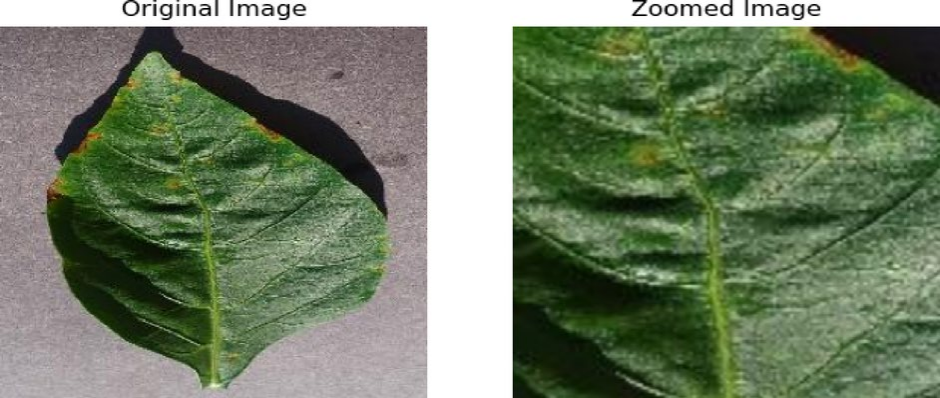
Data augmentation using zooming.

**Fig. 8:**
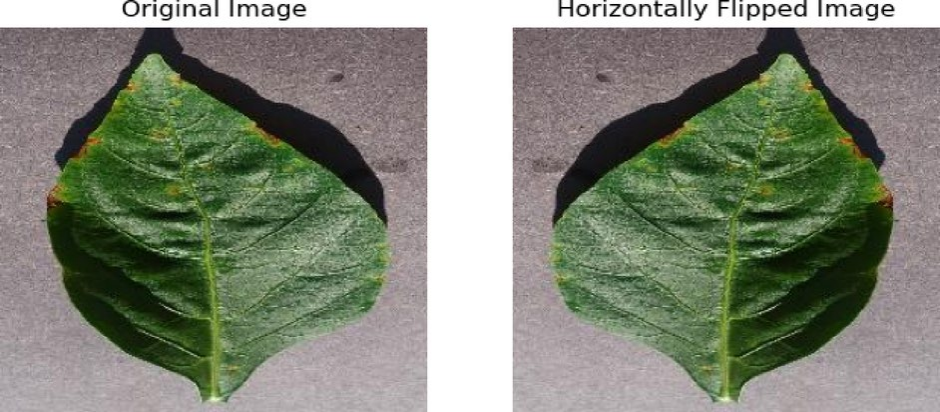
Data augmentation using horizontal flipping.

Figures 7 and 8 show the data augmentation results of zooming and horizontal flip, respectively. As shown in Fig. 7, zooming the original image helps the model to precisely detect the plant diseases. Figure 8 shows the transformation of the original image onto horizontally flipped image. This transformation is leading to train the model by diversifying the dataset and improving model robustness.

### Plant diseases detection results

The paper presents the system performance metrics of the ResNet50 model for plant diseases detection as: model accuracy, confusion matrix, and classification report.

Table 7 evaluates the accuracy of the ResNet50 model during training, validation, and testing the augmented 25,940 images using python programming on raspberry pi controller to classify one of the 11-plant classes. The confusion matrix reflects the ability of ResNet50 model to classify 11-classes plants in the 2,594 testing images of as a predicted classes compared to actual classes.

**Table 7:** Accuracy of ResNet50 model.

ResNet50 Model	Accuracy (%)	Loss
Training	99.9	5.8516 × 10^− 4^
Validation	99.69	0.0096
Testing	99.8	0.0244

The classification report indicates the evaluation of the performance metrics “precision, recall, and F1-score” for the 11-plant classes of the 2,594 testing images as listed in Table 8. High precision scores (close to 1.00) indicate that the model makes very few false positive errors across most classes and vice versa. High recall scores (close to 1.00) indicate that the model captures a high proportion of actual positive instances for each class and vice versa. The F1-score balances precision and recall, is consistently high across classes, showcasing the model’s overall robustness in classification.

**Table 8:** Classification report for the proposed ResNet50 model.

11-plant classes	Precision	Recall	F1-score
Pepper, bell__Bacterial_spot	1.00	0.99	1.00
Pepper, bell__healthy	0.99	1.00	0.99
Potato___Early_blight	1.00	1.00	1.00
Potato___Late_blight	1.00	1.00	1.00
Potato___healthy	1.00	1.00	1.00
Tomato___Bacterial_spot	0.98	1.00	0.99
Tomato___Early_blight	1.00	0.97	0.98
Tomato___Late_blight	0.99	0.99	0.99
Tomato___Tomato_Yellow_Leaf_Curl_Virus	1.00	1.00	1.00
Tomato___Tomato_mosaic_virus	1.00	1.00	1.00
Tomato___healthy	0.99	1.00	1.00
Accuracy	–	–	0.99
Macro Average	1.00	0.99	0.99
Weighted Average	1.00	0.99	0.99

The classification report results showed that the high accuracy, along with balanced precision, recall, and F1-scores across classes, underscores the effectiveness of our model in accurately classifying plant diseases. This performance is crucial for real-world applications, where accurate disease detection is paramount for effective crop management and yield optimization. The classification report has several evaluation metrics are calculated based on Equations from (4) to (7)^39^.

These metrics are essential in deep learning, particularly for classification models, as they help assess the performance of a model in various scenarios. Accuracy measures the overall correctness of the model by calculating the ratio of correctly predicted observations to the total number of observations. It provides a general measure of model performance but may not always be the best metric when dealing with imbalanced datasets. Precision focuses on the reliability of positive predictions by determining the proportion of correctly predicted positive cases out of all predicted positive cases. It is particularly important in situations where false positives need to be minimized, such as in medical diagnoses or fraud detection. Recall evaluates the model’s ability to correctly identify actual positive cases. It is calculated as the ratio of correctly predicted positive cases to the total number of actual positive cases. This metric is crucial when false negatives carry significant consequences, such as in disease detection or security applications. Finally, the F1-score serves as a balanced measure by taking the harmonic mean of precision and recall. It is especially useful for handling imbalanced datasets, where a high accuracy alone may not accurately reflect model performance. The F1-score ensures that both precision and recall are considered, making it a more comprehensive evaluation metric.

4 $$\:Precision=\frac{TP}{TP+FP}$$

5 $$\:Recall=\frac{TP}{TP+FN}$$

6 $$\:Accuracy=\frac{TP+TN}{TP+FP+TN+FN}$$

7 $$\:F1-score=2\times\:\frac{Precision\times\:Recall}{Precision+Recall}$$

**Fig. 9:**
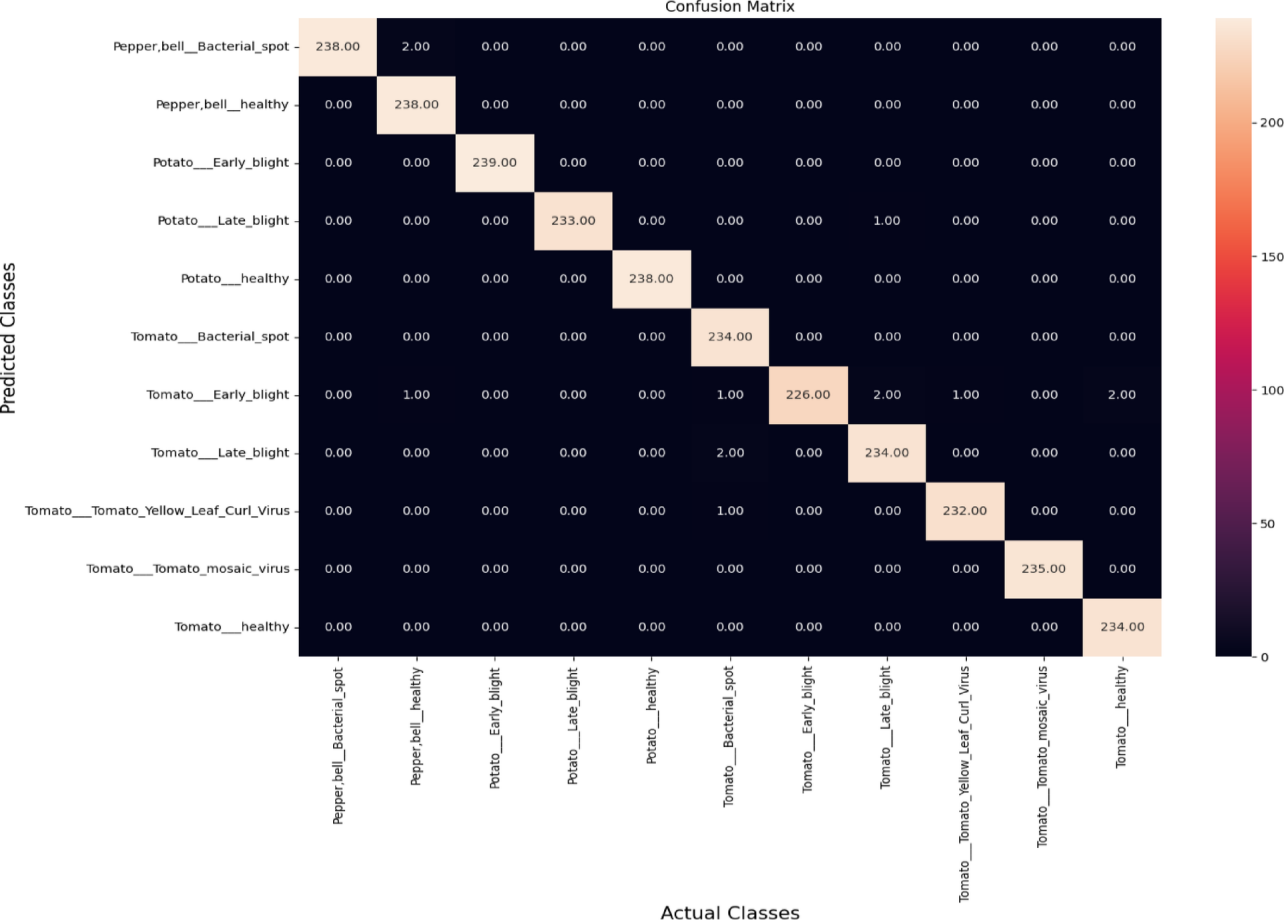
Confusion matrix of the ResNet50 model for plant diseases detection.

As seen in Fig. 9, the majority of classes exhibit diagonal dominance, indicating that the model’s predictions align well with the actual classes. On contrary, there are minimal off-diagonal elements of the dark colors that record minimum values of misclassifications. It is showed that the diseased classes “Pepper, bell__Bacterial_spot, Potato__Late_blight, Tomato__Tomato_Yellow_Leaf_Curl_Virus” record the perfect predictions (precision, recall, and F1-score of 1.00) and zero misclassification, reflecting the strong ability of ResNet50 model to distinguish these classes.

**Fig. 10:**
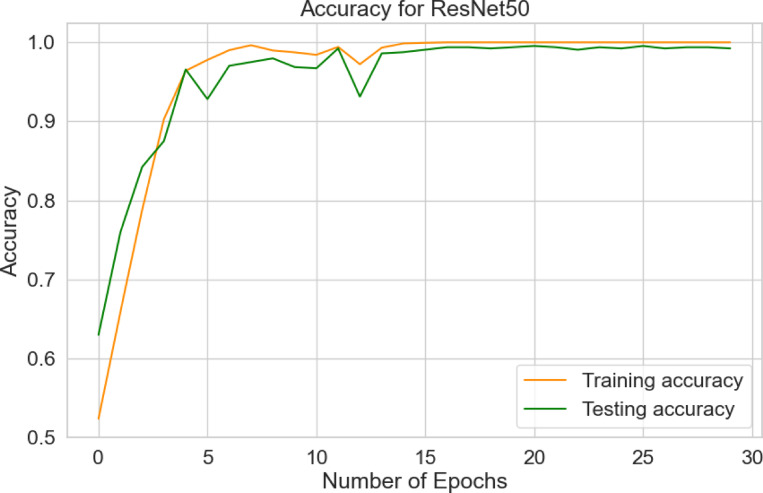
Accuracy for ResNet50.

**Fig. 11:**
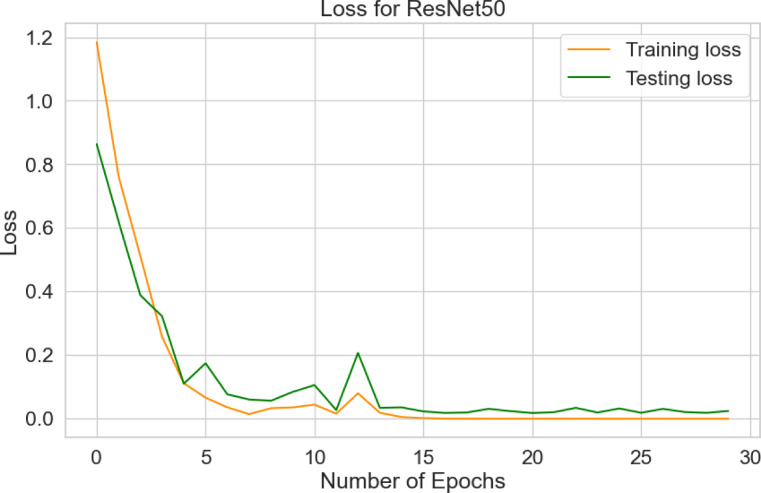
Loss for ResNet50.

Figure 10 shows the accuracy of training and testing for the ResNet50 model over 30 epochs. The orange training curve starts at 52.34% and reaches 100%. Similarly, the green testing curve begins at 62.97% and rises to 99.8%. Figure 11 shows the testing and training loss for the ResNet50. The training loss begins from 1.1838 and decreases to 0.00001 after 30 epochs, while the test loss starts at 0.8635 and down to 0.0244.

Finally, different DL models were trained, validated, and tested by using the same conditions of ResNet50 model such as data augmentation techniques, images size, image numbers, splitting ratio (70%, 20%, 10%), classes number, and configuration parameters. It is concluded that ResNet50 model outperforms other DL models due to their aspects discussed in the previous section. As listed in Table 9, the accuracy of ResNet50 model is higher than that in the VGG model, CNN model, and MOBILNET model. Table 10 lists the comparison study among different related works and the proposed work at the same conditions.

**Table 9:** Accuracy of different DL models compared to the proposed ResNet50 model.

DL models	Accuracy (%)
MobileNet	97.79
VGG	97.8
CNN	91.0
ResNet50	**99.8**

**Table 10:** Accuracy of some related works compared to the proposed AI-IoT smart agriculture Pivot.

Related work	Year	Employed technique	Accuracy (%)
Saikat Banerjee et al.^20^	2023	Presented a CNN model for detecting and identifying plant leaf diseases based on visual data to boost accuracy, generality, and the overall efficacy of training.	99.23
Xuewei Sun et al.^21^	2022	Proposed a convolutional neural network architecture FL-EfficientNet (Focal loss EfficientNet), which is used for multi-category identification of plant disease images. The experiment uses the public data set new plant diseases dataset (NPDD) and compares it with ResNet50, DenseNet169, and EfficientNet.	99.72
Santhana Krishnan et al.^22^	2023	Proposed an enhanced Convolution Neural Network based on VGG16 is used for potato leaf disease classification. The convolution layers of VGG16 along with the Inception and the SE block are used in this research for classification.	99.3
Nirmal Jothi Jerome et al.^23^	2023	Presented DL based Assessment-based Convolutional Neural Network (A-CNN) method to detect healthy and unhealthy plant leaves.	92
The proposed model	2024	An enhanced Convolution Neural Network method based on ResNet50 is proposed for detecting healthy and the diseases of plants like bell pepper, tomato and potato.	**99.8**

## Conclusion

This paper presents the plant diseases detection and treatment based a proposed AI-IoT Smart Agriculture Pivot for plant for solving the crop production and the lake of the food in the agriculture field. Furthermore, the paper presented different enhancement techniques and technologies and their related works, widely used for plant diseases detection. The paper introduced the most common agriculture devices as a solution for plant diseases treatment such as UAVs, robotics, and pivot. The proposed AI-IoT Smart Agriculture Pivot is architected, designed, implemented, programmed using python code, and controlled by Raspberry pi controller in order to achieve the plant diseases detection and treatment. The programming of the proposal includes data augmentation, and ResNet50 DL model for training, validation, and testing the augmented images from NPDD source. It is concluded that ResNet50 model scores a high accuracy during the classification of 11-plant classes compared to other DL models and outperforms other related works for plant diseases detection. Experimentally, the ResNet-50 achieved a testing accuracy of 99.8%, while F1-score, Recall, and Precision, for ResNet50 model were 99.91%, 99.92%, and 100%, respectively. A design of a mobile application is developed to provide farmers with expert advice and guidelines on plant disease detection and treatment methods. Further, the paper presents an automation of the spraying of fertilizers to treat identified plant diseases. Moreover, Raspberry pi controller can control the implemented hardware pilot for spraying the liquid on the farm land for the plant diseases treatment. In the future work, we look to improve the water irrigation process in the proposed AI-IoT smart agriculture pivot compared to the traditional central pivot for preserving water resources. The proposed pivot will develop the pest attack control and its treatment.

## Data Availability

The paper has a dataset available in the Kaggle repository https://www.kaggle.com/datasets/vipoooool/new-plant-diseases-dataset.
